# Estimating Caloric Intake per Breastfeeding Session in Infants: A Probabilistic Approach

**DOI:** 10.3390/nu17193136

**Published:** 2025-09-30

**Authors:** Ana Barrés-Fernández, José Vicente Arcos-Machancoses, Silvia Castillo-Corullón, Sergio Iniesta González, Maravillas Fullana-Tur, Susana Ferrando-Monleón

**Affiliations:** 1 Department of Pediatrics, Hospital Clínic Universitari de València, Valencia, Spain; barres_ana@gva.es (A.B.-F.); castillo_sil@gva.es (S.C.-C.);; 2 INCLIVA Biomedical Research Institute, Valencia, Spain; 3 Department of Pediatrics, Obstetrics and Gynecology, Universitat de València, Valencia, Spain

**Keywords:** infant nutrition, human milk composition, energy estimation

## Abstract

**Background/Objectives**: Accurate estimation of caloric intake from breastfeeding is essential for understanding infant nutrition during early life. However, most existing models rely on fixed assumptions and do not reflect the natural variability in feeding behaviors and human milk composition. This study aims to provide a realistic estimation of breast milk (BM) caloric intake throughout infancy using a probabilistic approach based on empirical data. **Methods**: A probabilistic model was developed using four variables: feeding frequency, volume per feeding, caloric density, and infant weight. Systematic reviews were conducted to inform the input values of the first three variables, and meta-analyses were performed when feasible. Infant weight was based on World Health Organization (WHO) growth standards. Variables were stratified by age and integrated into the model through appropriate probability distributions. Monte Carlo simulations were conducted to estimate caloric intake per kilogram of body weight, expressed both per day and per feeding, across all age groups. **Results**: The model showed a progressive decline in daily caloric intake per kilogram with age, consistent with decreasing feeding frequency and the introduction of complementary foods. In contrast, caloric intake per feeding increased with age. These findings align with WHO energy intake targets during exclusive breastfeeding and reflect expected physiological changes in infant growth and feeding behavior. **Conclusions**: This study provides a probabilistic framework for estimating BM caloric intake across infancy, accounting for interindividual and age-related variability. It offers a valuable research tool to support future studies on infant nutrition and feeding behavior using realistic, data-driven assumptions.

## 1. Introduction

Breastfeeding provides essential nutrients and energy for infant growth and development. The World Health Organization (WHO) recommends exclusive breastfeeding for the first six months, followed by continued breastfeeding up to two years of age or beyond [1].

However, accurately estimating caloric intake from breast milk (BM) remains challenging due to variability in feeding patterns, milk composition, and infant metabolic demands [2]. Methods such as the Deuterium Dilution Technique (DTM) and 24 h test weighing are reliable for estimating BM intake but have practical limitations: test weighing may interfere with natural breastfeeding, while DTM is time-consuming, costly, and difficult to implement in routine settings [2,3,4,5]. Additionally, these methods estimate total BM volume but do not provide detailed caloric intake per feeding session.

Reliable estimates of caloric intake are crucial for research. However, there is a lack of models providing age-specific caloric estimates per feeding. Most available approaches are based on fixed assumptions, such as a constant feeding frequency or average milk composition, which do not reflect the natural variability observed in breastfeeding practices [6,7]. To address this issue, we developed a probabilistic model that offers more realistic, individualized estimations of BM caloric intake, considering factors like breastfeeding volume, feeding frequency, milk composition, and infant weight. This approach allows for a flexible and reproducible framework that more accurately reflects real-life breastfeeding patterns. Recognizing the well-established benefits of breastfeeding and existing clinical guidelines, this model is intended solely as a research tool to explore the variability in caloric intake during breastfeeding, without aiming to guide clinical practice.

## 2. Materials and Methods

### 2.1. Model Objective and Design

A probabilistic model was developed in June 2025 to estimate the caloric contribution of individual breastfeeding sessions and the resulting BM daily caloric intake per kilogram of body weight in full-term infants aged 0 to 24 months.

The model integrated four key variables influencing caloric intake: BM caloric density, volume consumed per feeding, daily feeding frequency, and infant weight. The first three variables were derived from systematic literature reviews and, where applicable, meta-analyses, whereas infant weight was directly obtained from the WHO Child Growth Standards [8].

The primary objective of the model was not to provide individualized clinical recommendations but rather to generate realistic, population-based estimates that can support nutritional assessments and future research on infant feeding.

### 2.2. Age Stratification and Data Transformation

Infant age was divided into intervals: 0–1, 1–3, 3–6, 6–9, 9–12, 12–18, and 18–24 months. The neonatal period (0–1 month) was analyzed separately to account for rapid developmental changes and the distinct profile of milk composition during the first weeks of life [9,10]. From 1 month onward, 3-month intervals were used in the first year, and broader intervals (12–18 and 18–24 months) in the second year to align with available data and primary sources. Age assignment was based on completed months to avoid overlap.

### 2.3. Systematic Review and Meta-Analysis

To generate evidence-based input parameters for the probabilistic model, we performed three independent systematic reviews targeting the main determinants of BM caloric intake: (i) caloric density, (ii) volume per feeding, and (iii) daily breastfeeding frequency.

The reviews followed the PRISMA 2020 framework. Searches were conducted in PubMed from January 2005 to December 2025. We complemented the process using Elicit (Ought, San Francisco, CA, USA), an AI-assisted tool for evidence synthesis; however, all references were screened and verified manually by two independent reviewers, and AI was used only as a complementary tool [11]. Due to the strict inclusion and exclusion criteria, the number of eligible studies was limited.

#### 2.3.1. Eligibility Criteria

- Inclusion: original observational or interventional studies reporting quantitative data on the target variables; healthy, full-term infants aged 0–24 months; and results stratified by infant age or time since birth.
- Exclusion (general):
  1. Narrative/review articles, editorials, protocols, or meta-analyses without primary data.
  2. Preterm infants only (unless data for term infants were reported separately).
  3. Out of time range (<2005).
  4. Non-human or animal studies.
  5. Non-applicable populations (e.g., cohorts with severe maternal malnutrition, rare diseases, or humanitarian crisis contexts).
  6. Studies analyzing donor human milk, due to compositional changes introduced by pasteurization that may alter caloric density and nutrient content [12].
  7. Other: abstracts without usable data, duplicates, or incomplete reports.

Additionally, variable-specific exclusion criteria were applied:

- Caloric density: studies not reporting caloric density or macronutrient content of BM, data not in quantitative format (mean and standard deviation), or those focused solely on biochemical/methodological analyses without cohort-level values.
- BM volume per feeding: studies not reporting BM volume, not providing data per feeding, lacking quantitative format (no means/medians), or using only indirect methods (e.g., maternal recall without test weighing or D_2_O).
- Feeding frequency: studies not reporting the number of feeds per day in an applicable format (e.g., only weekly frequency, feeding duration without daily count, or broad categorical groups without averages).

#### 2.3.2. Study Selection and Extraction

Titles and abstracts were screened independently by two reviewers, with disagreements resolved by consensus. For each included study, we extracted: study design, country, sample size, infant age, measurement method, and quantitative results. All variables were standardized to common units (kcal/dL, mL/feed, feeds/day).

Detailed search strategies for PubMed and Elicit, including all Boolean operators and filters applied, are provided in Supplementary Materials Table S1.

#### 2.3.3. Meta-Analysis

A meta-analysis was conducted for variables meeting these criteria: (1) data from at least two studies; (2) reporting means and standard deviations (mean ± standard deviation); and (3) age stratification consistent with the model. Eligible studies were synthesized using MetaAnalysisOnline.com, applying a random-effects model with inverse variance weighting to generate pooled means and 95% confidence intervals (CIs). Statistical heterogeneity was assessed using Cochran’s Q test, Tau^2^, and the *I*^2^ statistic. Forest plots were generated for visual inspection [13].

### 2.4. Infant Weight as Input Parameter

Unlike the other variables, infant weight was not derived from a literature review but extracted directly from the WHO Child Growth Standards. We selected the 50th percentile (P50) for girls and boys as the most representative indicator of typical growth in full-term infants [8]. This approach avoids the influence of outliers inherent in mean-based values and reflects the median expected weight in the international reference population. To ensure consistency and account for sex-related growth differences, we consistently reported the range defined by the P50 of girls (lower bound) and boys (upper bound) for each age group. This methodology ensured internal coherence across input variables and alignment with internationally recognized anthropometric standards.

### 2.5. Probability Distributions and Simulation

A second-degree Monte Carlo simulation (35,000 iterations; 5000 per age group) was performed using XLRisk^®^ for Microsoft Excel (2025, Redmond, WA, USA) [14]. Convergence and stability of the simulations were verified by monitoring the stabilization of mean and percentile values across successive iterations, ensuring robust estimates. Each input variable was assigned a probability distribution based on its statistical nature and the characteristics of the available data:

- **Feeding frequency** (feeds/day) was modeled using a triangular distribution, suitable for variables with limited data and defined minimum, maximum, and most likely values.
- **Volume per feeding** (mL/feed) was modeled using Project Evaluation and Review Technique (PERT) distributions, which account for asymmetry and wide individual variability while limiting the influence of outliers. These are appropriate for continuous variables when expert-informed minimum, most likely, and maximum values are available, and normality cannot be assumed.
- **Caloric density** (kcal/dL) was modeled using either a normal or a PERT distribution, depending on the heterogeneity observed in the meta-analysis. For age groups with low to moderate heterogeneity, a normal distribution was applied using the pooled mean and standard deviation (from the 95% confidence interval). For groups with high heterogeneity, a PERT distribution was used, with CI limits defining the range and the pooled mean as the most likely value. This approach enabled accurate representation of both consistent and variable results while minimizing outlier effects.
- **Infant weight** was further bounded using a uniform distribution, with the lower and upper limits defined by the 50th percentile values for girls and boys, respectively, according to the WHO Child Growth Standards. This choice constrained values to a biologically plausible range, assuming equal probability across the interval in the absence of more detailed distributional data.

To facilitate interpretation, simulated outcomes were categorized into three levels of caloric intake: low (LCI, Q1), medium (MCI, Q2), and high caloric intake (HCI, Q3), providing a structured framework for evaluating variability in nutritional intake across different age groups.

## 3. Results

### 3.1. Results of the Systematic Review and Meta-Analysis

#### 3.1.1. Breast Milk Caloric Density

**Figure 1:**
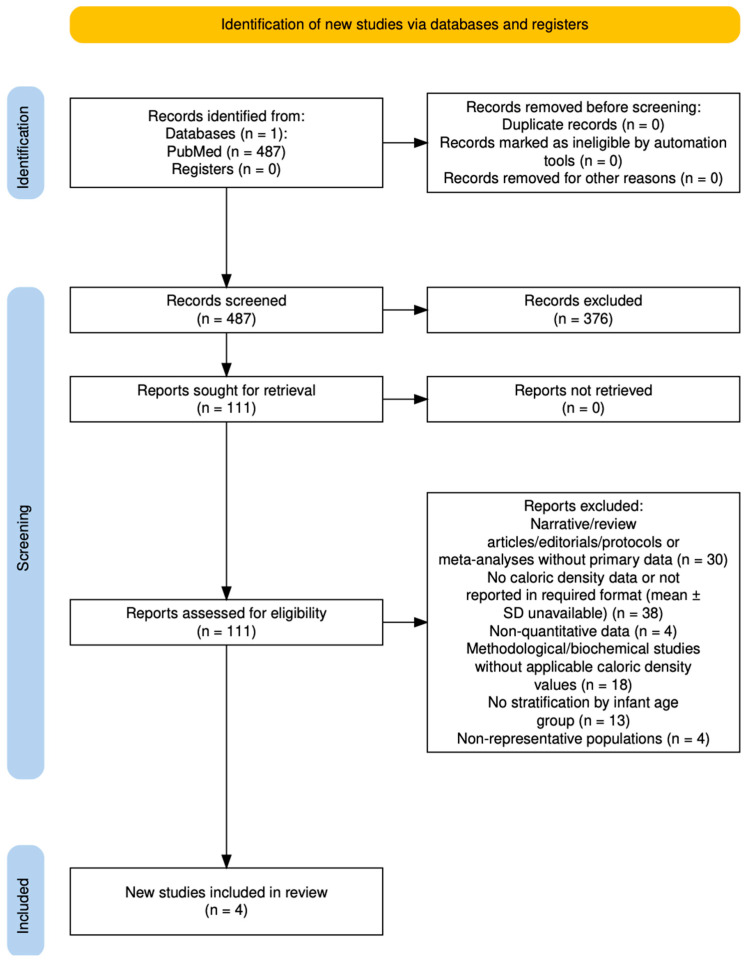
PRISMA 2020 flow diagram of study selection for breast milk caloric density (2005–2025).

The systematic review identified 487 records in PubMed, of which 4 studies met the inclusion criteria after screening and full-text assessment (Figure 1). In parallel, the process was complemented with Elicit, which retrieved 8 candidate studies, of which 2 additional articles fulfilled the eligibility criteria. Thus, a total of 6 studies were included in the final synthesis (Table 1).

Studies by Czosnykowska-Łukacka et al. and Ongprasert et al. contributed exclusively to the 9–12 months age group, as their data for earlier months were aggregated into overly broad categories and therefore excluded from the 0–9 months analysis [15,16].

Reasons for exclusion of PubMed-identified records are detailed in Supplementary Table S2, while the screening process for Elicit-derived articles is provided in Supplementary Table S3.

The meta-analysis results are presented in Supplementary Tables S8–S14. High heterogeneity was observed in several age groups—particularly 0–1 and 9–12 months—while the 6–9-month group showed no heterogeneity (*I*^2^ = 0%). This meta-analysis was performed exclusively to generate standardized, age-specific inputs for the probabilistic model.

**Table 1:** Summary of studies identified through PubMed and Elicit search on breast milk caloric density in full-term infants (2005–2025).

Source	Study (Author, Year)	Country	Design	Sample Size	Age Range Covered	Method Used	Included in Meta-Analysis
PubMed and Elicit	Saarela et al., 2005 [17]	Finland	Longitudinal cohort	483	1 week–6 months	Chemical/enzymatic methods	Yes
Elicit	Chang et al., 2015 [10]	South Korea	Observational cohort	2000+	0–9 months	FT-IR	Yes
PubMed and Elicit	Grote et al., 2016 [18]	Multicountry	Cohort	174	1–6 months	GC, HPLC	Yes
PubMed and Elicit	Czosnykowska-Łukacka et al., 2018 [15]	Poland	Mixed methods	137	0–24 months	MIRIS	Partially (9–24 months)
PubMed and Elicit	Fischer Fumeaux et al., 2019 [9]	Switzerland	Longitudinal cohort	61	0–3 months	MIRIS	Yes
Elicit	Ongprasert et al., 2020 [16]	Thailand	Cross-sectional	184	6–24 months	Colorimetric	Partially (9–24 months)

Abbreviations: FT-IR: Fourier-transform infrared spectroscopy; GC: gas chromatography; HPLC: high-performance liquid chromatography; MIRIS: mid-infrared human milk analyzer. Source: Created by the authors based on data extracted through a systematic review of PubMed and complemented with Elicit (Ought, San Francisco, CA, USA) [11]. Study screening and data extraction were conducted independently by two reviewers. Data adapted from Saarela et al., 2005, Fischer Fumeaux et al., 2019; Chang et al., 2015; Czosnykowska-Łukacka et al., 2018; Ongprasert et al., 2020; Grote et al., 2016 [9,10,15,16,17,18].

#### 3.1.2. Volume per Feeding

**Figure 2:**
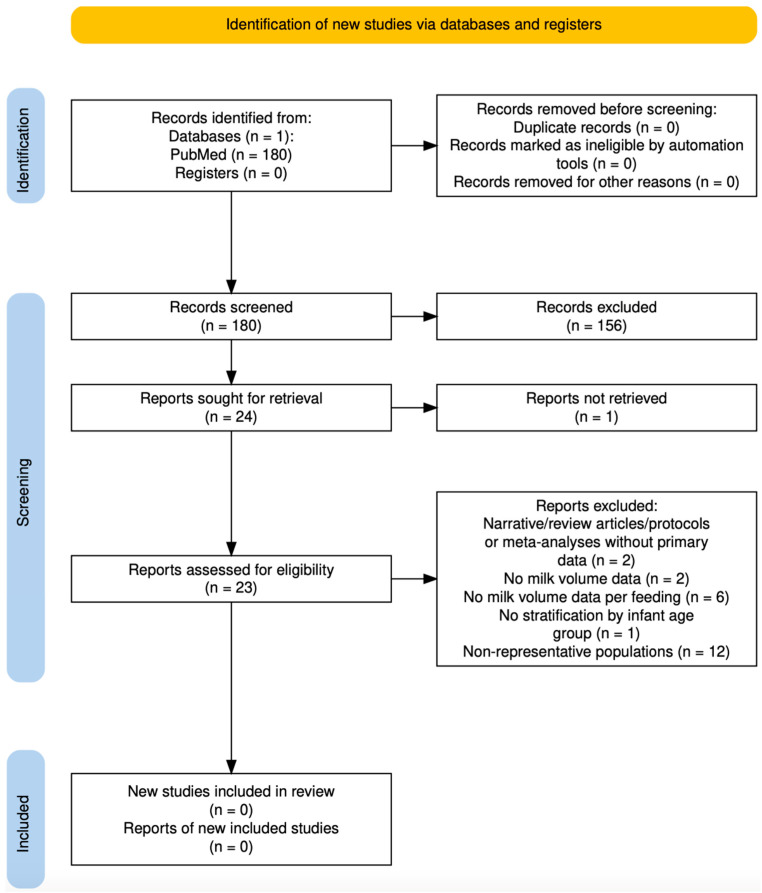
PRISMA 2020 flow diagram of study selection for breast milk volume per feeding (2005–2025).

The systematic review identified 180 records in PubMed, of which 24 were selected for full-text screening. After eligibility assessment, none fulfilled the predefined inclusion criteria (Figure 2). The main reasons for exclusion were absence of BM volume data per feeding, lack of stratification by infant age, studies providing only daily volume or indirect estimations, and non-representative populations. In addition, a search was conducted with Elicit, which also yielded no eligible records. Details of the exclusion criteria applied to PubMed-identified records are presented in Supplementary Table S4, whereas Supplementary Table S5 summarizes the screening outcomes of articles retrieved through Elicit.

Although excluded due to insufficient stratification by infant age, two studies by Kent and colleagues are noteworthy as they represent the only available evidence reporting BM volume per feeding in infants under 6 months [19,20]. All measurements were expressed in grams but can be considered approximately equivalent to milliliters, as human milk density is ~1.03 g/mL [21]. Using test-weighing, Kent et al. (2006) observed a mean intake of 76.0 ± 12.6 g per feed (range: 0–240 g), with an inverse relationship between daily feeding frequency and average volume per feed (r^2^ = 0.442, *p* < 0.001) [19]. The average per meal was 101.4 ± 15.6 g, with increasing maximum intake as age advanced between 4 and 26 weeks [19].

In a subsequent study, Kent et al. (2013) reported a median intake of 106 mL per feed at 1 month, with a maximum of 162 mL [20]. Median milk intake increased by 4.1 mL/week during early lactation, while maximum intake increased by 4.4 mL/week. However, in later lactation no significant changes were observed. The total 24 h intake from 1 to 6 months averaged 808 g (SD 192; range 463–1370 g) [20].

Despite not meeting the predefined inclusion criteria, these studies informed the plausibility checks of the probabilistic model, as they provide the only direct measurements of BM intake per feeding session in early infancy.

For older infants, no direct per-feed data were available, and the literature instead provided only daily intakes. Given this limitation, we complemented the synthesis with indirect sources. Several studies reported 24 h milk volumes, typically measured by test weighing or DTM. Reported values ranged from ~600–720 mL/day during early lactation to ~450–500 mL/day by 12 months. These results are broadly consistent with international references [22,23].

While these three studies formed the basis of our estimates, we supported the plausibility of the modeled intake ranges using WHO feeding recommendations for formula-fed infants, which suggest a gradual increase from 60 to 150 mL/kg/day during the early postnatal period [24]. Although not directly applicable to breastfed infants, these values provide a physiological reference framework consistent with growth and feeding patterns. Based on this, and given the observed variability across studies, we selected broad volume ranges for each age group to be integrated into the simulation model. 

#### 3.1.3. Breastfeeding Frequency

**Figure 3:**
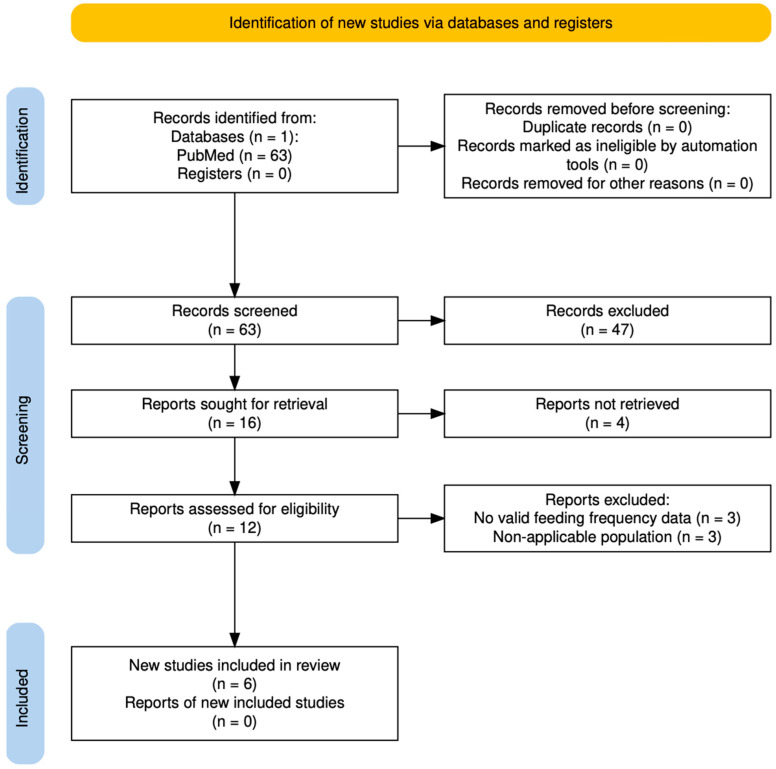
PRISMA 2020 flow diagram of study selection for breastfeeding frequency (2005–2025).

The systematic review identified 63 records in PubMed, of which 6 studies met the inclusion criteria after screening and full-text assessment (Figure 3). In parallel, the process was complemented with Elicit, which retrieved 6 candidate studies; 3 were excluded and 1 overlapped with PubMed, resulting in 3 additional eligible articles. Thus, a total of 9 studies were included in the final synthesis (Table 2). Reasons for exclusion of PubMed-derived records are detailed in Supplementary Table S6, while the screening process for Elicit-derived articles is shown in Supplementary Table S7.

Due to the limited availability of standardized, age-specific data and heterogeneous reporting formats, no meta-analysis was performed. Instead, we extracted medians and interquartile ranges (IQRs) from the selected studies to define plausible frequency intervals for each age group.

For the 0–1 month group, Kent et al. (2013) reported an average of 7.6 feeds/day at one month, with a gradual decline of –0.2 feeds per week and an increase in the longest interval between feeds [20]. Kent et al. (2006) described a broad range of 6 to 18 feeds/day in exclusively breastfed infants aged 1–6 months [19]. Similarly, Huang et al. y Saki et al. documented 10–14 feeds/day in newborns aged 0–28 days, while Chen et al. reported a median of 8 feeds/day (IQR: 7–10) in the first two weeks of life. [27,28,30]. Based on these data, a range of 8–14 feeds/day was estimated for the 0–1 month group.

For 1–12 months, data from the WHO Multicenter Growth Reference Study provided medians and IQRs across several countries: 9 feeds/day (IQR: 7–11) at 3 months, 8 feeds/day (IQR: 6–10) at 6 months, 7 feeds/day (IQR: 5–9) at 9 months, and 6 feeds/day (IQR: 4–8) at 12 months [26]. These values were consistent with other reports, such as Thomas et al. (10.3 feeds/day at 1–3 months), Ongprasert et al. (7.0 ± 2.2 feeds/day at 6–11 months), and Gridneva et al. (8.1 ± 1.4 feeds/day at 5 months; 5.4 ± 1.2 at 9 months) [25,29,31]. For modeling purposes, these IQRs were slightly adapted into simplified intervals to harmonize across studies and ensure consistent input categories. Accordingly, the following input intervals were defined:

- 1–3 months: 7–10 feeds/day
- 3–6 months: 6–9 feeds/day
- 6–9 months: 5–8 feeds/day
- 9–12 months: 4–7 feeds/day

For the second year of life, given the scarcity of age-specific studies and the increasing variability associated with prolonged lactation, broader ranges were proposed. Ongprasert et al. reported 4.9 ± 1.8 feeds/day at 12–17 months and 3.8 ± 2.1 at 18–23 months, while Gridneva et al. found 4.4 ± 1.9 feeds/day at 12 months [29,31]. Based on these observations, the following intervals were considered:

- 12–18 months: 2–6 feeds/day
- 18–24 months: 2–4 feeds/day

The final input values for each group are summarized in Table 3.

#### 3.1.4. Final Input Parameters for the Probabilistic Analysis

All probabilistic input parameters employed in the simulation model for estimating BM caloric intake across age groups—used for the Monte Carlo simulations performed with XLRisk^®^ for Microsoft Excel (2025, Redmond, WA, USA) [14]—are detailed below. Table 3 summarizes the selected input values extracted from the literature, while Table 4 presents the probability distributions applied for each parameter.

### 3.2. Probabilistic Model Results

**Figure 4:**
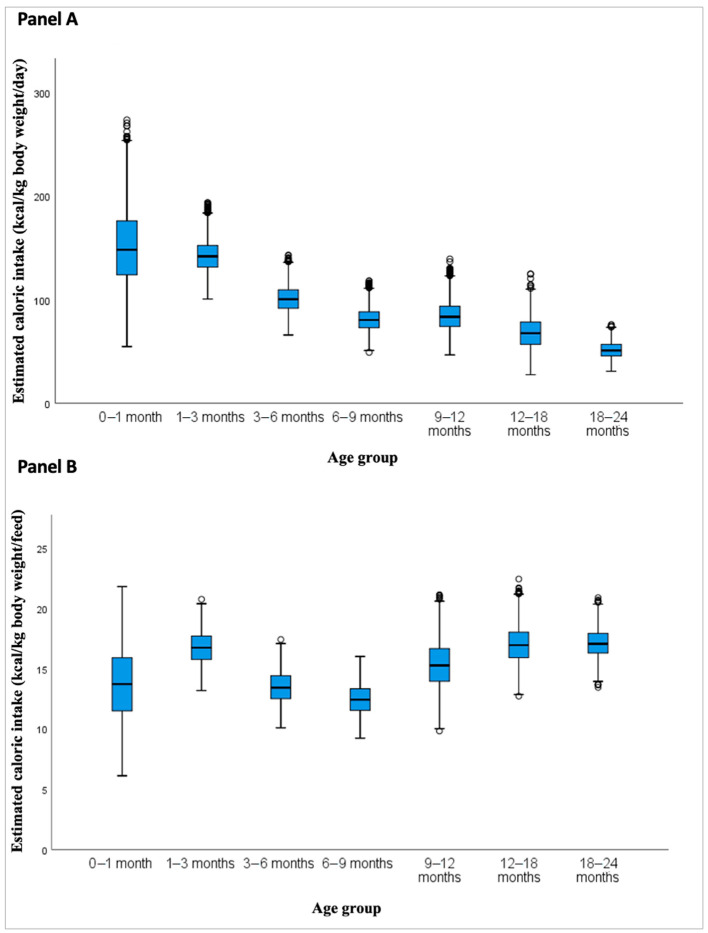
Estimated caloric intake from breastfeeding across infancy based on probabilistic simulations. (**A**) Simulated caloric intake per day (kcal/kg body weight/day) by age group. (**B**) Simulated caloric intake per feeding (kcal/kg body weight/feed) by age group.

As shown in Table 5 and Figure 4A, the estimated daily caloric intake from BM per kilogram of body weight progressively declined with age. The mean intake decreased from 150.8 kcal/kg/day in the 0–1 month group to 51.3 kcal/kg/day in the 18–24-month group. A slight increase was observed in the 9–12-month group (84.3 kcal/kg/day) compared to 6–9 months (80.8 kcal/kg/day), accompanied by a wider interquartile range and a higher standard deviation (SD = 14.2 kcal/kg/day), indicating increased variability in this subgroup.

Table 5 and Figure 4B illustrates caloric intake per feeding (kcal/kg/feed), which generally increased with age, rising from 13.7 kcal/kg/feed in the first month to 17.1 kcal/kg/feed in the oldest group. However, the trend was not linear. A distinct peak was observed at 1–3 months (16.8 kcal/kg/feed), followed by a transient decrease in the 3–6-month group (13.5 kcal/kg/feed), with intake values increasing again from 6 months onward.

For each age group, the results were also presented according to low (LCI, Q1), medium (MCI, Q2), and high caloric intake (HCI, Q3), which allows for a stratified interpretation of variability across the simulated distributions.

## 4. Discussion

Accurate estimation of caloric intake from breastfeeding is a key challenge in infant nutrition research, yet few tools offer age-specific values per feeding. Existing models often rely on arbitrary fixed assumptions that fail to reflect the natural variability of breastfeeding patterns and may lead to inaccurate estimations, particularly in research contexts requiring detailed intake modeling [6,7].

Our probabilistic approach offers a more realistic alternative. By integrating diverse data sources and applying probabilistic simulations, we modeled caloric intake from BM using distributions that reflect variability in feeding volume and frequency, milk composition, and infant weight. This allowed us to simulate realistic feeding scenarios across age groups, better representing actual clinical situations.

Systematic literature reviews were conducted to define the input parameters of the model, but caloric density was the only variable suitable for meta-analysis, allowing for the generation of evidence-based probability distributions across age groups. In contrast, feeding frequency and volume per feeding were informed by published ranges, as they did not meet criteria for quantitative synthesis.

The model estimates a clear decreasing trend in daily caloric intake per kilogram of body weight with advancing age: from 150.8 kcal/kg/day in the 0–1 month group to 51.3 kcal/kg/day at 18–24 months. This decline is consistent with physiological changes during development, such as reduced metabolic demands per kilogram and decreased breastfeeding frequency over time. Conversely, caloric intake per feeding increases with age, rising from 13.7 kcal/kg/feed in the first month to 17.1 kcal/kg/feed in the oldest group, reflecting the rise in BM energy density and greater intake per session [9,10,15,16,18].

The 9–12-month group showed a modest increase in estimated intake (84.3 kcal/kg/day) with greater variability (SD = 14.2). This may relate to the particularly high caloric density estimate (76.7 kcal/dL), based on few studies with high heterogeneity (*I*^2^ = 98.7%). While plausible, this finding should be interpreted cautiously.

In addition, the higher caloric intake per kilogram of body weight per feeding estimated for the 1–3-month group likely represents a physiological peak driven by increased feeding volume, relatively low body weight, and slightly higher BM caloric density [19,20]. These factors, derived from our systematic review, may explain the highest modeled value observed across infancy, though the estimate should be interpreted considering the limitations of the source data.

Our results (Table 5) are generally consistent with the WHO energy requirement estimates, which range from 115 to 120 kcal/kg/day at 0–3 months, 95–105 kcal/kg/day at 3–6 months, 85–95 kcal/kg/day at 6–12 months, and 80–85 kcal/kg/day at 12–24 months [32]. While the model outputs for infants aged 0–6 months align well with these recommendations, estimates for older infants appear to follow a natural decline. This trend likely reflects the progressive reduction in breastfeeding frequency observed with age, as described in the WHO Multicenter Growth Reference Study and supported by other longitudinal reports [25,29,31], together with the introduction of complementary feeding after 6 months, which contributes to changes in total energy intake during the second half of infancy [1,32].

Our study offers several advantages over previous models that relied on fixed assumptions, such as Weisgerber et al. in 2013 [6]. Their model used a predetermined number of daily feedings and assumed fixed BM caloric values (82 kcal/kg/day up to 9 months and 53 kcal/kg/day up to 12 months) without strong supporting evidence [6]. In contrast, our model incorporates flexible distributions for feeding frequency, BM caloric density, feeding volumes, and infant growth patterns, capturing natural variability and providing a more realistic estimation of individual intake across different age groups.

The categorization into low (LCI), medium (MCI), and high (HCI) caloric intake provides a structured framework to model perceived feeding scenarios. This approach facilitates the interpretation of individual variability across age groups and supports more refined analyses of breastfeeding patterns in research on infant energy intake.

As a practical reference, our model estimates caloric intake (kcal/kg body weight per feeding and per day) across infancy, serving as a practical reference when direct measurement is not possible (Table 5). It may also be applied retrospectively in existing cohorts to estimate caloric intake from breastfeeding, for example, in studies that sought to relate energy intake to clinical outcomes. In addition, it can contribute to epidemiological research by providing standardized estimates of energy intake across age groups, which are often missing in large population-based datasets. Beyond nutrition, it may also provide value to clinical pharmacology modeling efforts related to lactation-associated drug exposure.

Detailed inputs and probability distributions are provided in Table 4, allowing reproducibility with tools like XLRisk^®^ for Microsoft Excel. The model is adaptable to specific contexts, such as preterm infants or populations affected by malnutrition, by adjusting parameters like age, weight, and milk composition, making it useful for both population-based analyses and hypothesis-driven research.

Nevertheless, there are several important limitations: only BM caloric density met meta-analysis criteria, while other variables relied on literature-based ranges, reflecting the scarcity of primary studies with stratified data by age. Furthermore, the model does not consider maternal nutritional status, child health, or socioeconomic and cultural diversity, since most input data come from studies conducted in high- and middle-income countries. These factors may influence feeding patterns in specific subgroups, and studies including data from more diverse settings are needed to broaden applicability [7]. The model also relied on WHO growth standards to define infant weight, which are widely accepted and derived from breastfed populations but may not fully capture variability in body composition across different regions [8]. Incorporating alternative growth data could help extend applicability. In addition, validation against empirical data from prospective cohorts would provide an extra layer of confirmation and support the broader use of the model.

## 5. Conclusions

This study presents the first published probabilistic model to estimate BM caloric intake per feeding and per day in healthy, full-term infants. By integrating data from systematic reviews and simulating intake patterns across age groups, it offers a realistic and flexible framework for assessing energy intake in population-based research. The model captures the natural variability of breastfeeding and supports future studies in the field of infant nutrition. In particular, it may help generate standardized estimates for epidemiological analyses or serve as a complementary approach in retrospective studies, always within research rather than a clinical context.

## Figures and Tables

**Table 2:** Summary of studies identified through PubMed and Elicit search on breastfeeding frequency in full-term infants (2005–2025).

Source	Study (Author, Year)	Country	Design	Sample Size	Age Range Covered	Method Used	Included in Analysis
PubMed	Thomas et al., 2005 [25]	USA	Longitudinal cohort	37	4–10 weeks	Structured maternal interviews	Yes
Elicit	Study Group, 2006 [26]	Multicountry	Longitudinal cohort	1743	0–24 months	24 h recall, structured interview	Yes
PubMed and Elicit	Kent et al., 2006 [19]	Australia	Cross-sectional observational	71	1–6 months	Direct observation (not explicitly stated)	Yes
Elicit	Saki et al., 2013 [27]	Iran	Longitudinal cohort	287	0–6 months	Structured maternal interviews	Yes
Elicit	Kent et al., 2013 [20]	Australia	Longitudinal cohort	52	0–6 months	Direct observation (not explicitly stated)	Yes
PubMed	Chen et al. 2015 [28]	Taiwan	Longitudinal cohort	98	0–2 weeks	Direct observation	Yes
PubMed	Gridneva et al., 2018 [29]	Australia	Longitudinal cohort	20	0–12 months	Direct observation and structured maternal interviews	Yes
PubMed	Huang et al., 2020 [30]	Taiwan	Longitudinal cohort	65	1–28 days	Structured maternal interviews	Yes
PubMed	Ongprasert et al., 2025 [31]	Northern Thailand	Cross-sectional observational	1122	6–24 months	Structured maternal interviews	Yes

Abbreviations: USA: United States of America. Source: Created by the authors based on data extracted through a systematic review of PubMed and complemented with Elicit (Ought, San Francisco, CA, USA) [11]. Study screening and data extraction were conducted independently by two reviewers. Data adapted from Thomas et al., 2005; Study Group, 2016; Kent et al., 2006; Saki et al. 2013; Kent et al., 2013; Chen et al. 2015; Gridneva et al., 2018; Huang et al., 2020; Ongprasert et al., 2025 [19,20,24,25,26,27,28,29,30,31].

**Table 3:** Selected input values used for the probabilistic model of caloric intake from breastfeeding, stratified by age group, and corresponding data sources.

Age (Months)	Feedings per Day	Caloric Density (kcal/dL)	Volume per Feeding (mL)	Median Weight (kg, WHO Growth Charts)
0–1	8–14	63.70 [62.10; 65.31]	30–110	3.2–3.3
1–3	7–10	64.95 [62.52; 67.38]	100–150	4.7–5
3–6	6–9	61.66 [60.18; 63.15]	110–180	6.3–7
6–9	5–8	61.74 [60.19; 63.29]	120–200	7.6–8.3
9–12	4–7	76.73 [55.16; 98.30]	140–200	8.5–9.2
12–18	2–6	83.65 [74.32; 92.97]	160–240	9.5–10.2
18–24	2–4	90.43 [81.76; 99.10]	180–240	10.8–11.4
Reference	Thomas et al., 2005 [25]; Study Group, 2006 [26], Kent et al. 2006 [19]; Saki et al., 2013 [27]; Kent et al., 2013 [20]; Chen et al., 2015 [28]; Gridneva et al., 2018 [29]; Huang et al., 2020 [30]; Ongprasert et al., 2025 [31]	Saarela et al., 2005 [17]; Chang et al., 2015 [10]; Grote et al., 2016 [18]; Czosnykowska-Łukacka et al., 2018 [15]; Fischer Fumeaux et al. 2019 [9]; Ongprasert et al., 2020 [16]	Olga et al., 2023 [22]; Kent et al. 2006 [19]; Kent et al. 2013 [20]; Mohr et al., 2023 [23]; WHO, 2009 [24].	Child growth standards (WHO) [8]

Abbreviations: WHO: World Health Organization. Source: Data adapted from Thomas et al., 2005; Study Group, 2006; Kent et al., 2006; Saki et al., 2013; Kent et al., 2013; Chen et al., 2015; Gridneva et al., 2018; Huang et al., 2020; Ongprasert et al., 2025; Saarela et al., 2005; Chang et al., 2015; Grote et al., 2016; Czosnykowska-Łukacka et al., 2018; Fischer Fumeaux et al. 2019; Ongprasert et al., 2020; Olga et al., 2023; Mohr et al., 2023; WHO, 2009; and Child growth standards [8,9,10,15,16,17,18,19,20,22,23,25,26,27,28,29,30,31].

**Table 4:** Input parameters and probability distributions used for the probabilistic model of caloric intake from breastfeeding, stratified by age group.

Age (Months)	Feedings Per Day	Caloric Density (kcal/dL)	Volume Per Feeding (mL)	Median Weight (kg, WHO Growth Charts)
Distribution	Triangular	PERT */Normal **	PERT	Uniform
0–1	a: 8; m: 11; b: 14	* a: 62.10; m: 63.70; b: 65.3	a: 30; m: 70; b: 110	a: 3.2; b: 3.3
1–3	a: 7; m: 8.5; b: 10	* a:62.52; m:64.95; b:67.38	a: 100; m: 125; b: 150	a: 4.7; b: 5.0
3–6	a: 6; m: 7.5; b: 9	** μ: 61.66; σ: 0.76	a: 110; m: 145; b: 180	a: 6.3; b: 7.0
6–9	a: 5; m: 6.5; b: 8	** μ: 61.74; σ: 0.79	a: 120; m: 160; b: 200	a: 7.6; b: 8.3
9–12	a: 4; m: 5.5; b: 7	* a: 55.16; m: 76.73; b: 98.30	a: 140; m: 180; b: 200	a: 8.5; b: 9.2
12–18	a: 2; m: 4; b: 6	* a: 74.32; m: 83.65; b: 92.97	a: 160; m: 200; b: 240	a: 9.5; b: 10.2
18–24	a: 2; m: 3; b: 4	* a: 81.76; m: 90.43; b: 99.10	a: 180; m: 210; b: 240	a: 10.8; b: 11.4

Abbreviations: PERT: Project Evaluation and Review Technique; WHO: World Health Organization. Variables were modeled in XLRisk^®^ using Triangular, PERT, and Normal functions. Parameters: a: minimum value; m: most likely value; b: maximum value; μ: mean; σ: standard deviation. * and ** refers to the type of distribution as indicated in the column subtitle (PERT *, and Normal **).

**Table 5:** Estimated caloric intake from breast milk per feeding and per day (kcal/kg body weight) by age group.

	Age (Months)	0–1	1–3	3–6	6–9	9–12	12–18	18–24
Mean	Kcal/kg/feed	13.71	16.75	13.46	12.43	15.32	16.99	17.11
	Kcal/kg/day	150.80	142.34	100.92	80.83	84.28	67.94	51.33
SD	Kcal/kg/feed	2.96	1.33	1.31	1.23	1.92	1.49	1.16
	Kcal/kg/day	36.88	15.27	12.77	11.13	14.17	15.03	7.77
LCI (Q1)	Kcal/kg/feed	11.49	15.76	12.51	11.54	13.95	15.92	16.30
	Kcal/kg/day	124.11	131.53	91.68	72.89	74.14	56.91	45.73
MCI (Q2)	Kcal/kg/feed	13.71	16.74	13.42	12.43	15.27	16.96	17.06
	Kcal/kg/day	148.33	141.98	100.41	80.46	83.42	67.64	51.01
HCI (Q3)	Kcal/kg/feed	15.91	17.71	14.42	13.33	16.67	18.02	17.93
	Kcal/kg/day	176.09	152.49	109.56	88.27	93.68	78.45	56.90

Based on second-order Monte Carlo simulations (5000 iterations per age group) using probabilistic inputs for feeding frequency, volume, caloric density, and infant weight. Values are expressed as kcal per kg of infant body weight per feed or per day. LCI, MCI, and HCI correspond to the first (Q1), second or median (Q2), and third quartiles (Q3), respectively. Abbreviations: SD: standard deviation. LCI: low caloric intake; MCI: medium caloric intake; HCI: high caloric intake.

## Data Availability

All data supporting the findings of this study are contained within the article and its Supplementary Materials.

## Supplementary Materials

The following supporting information can be downloaded at https://www.mdpi.com/article/10.3390/nu17193136/s1: Supplementary Table S1: Search strategies used for the systematic reviews. Date of search Aug 2025; Supplementary Table S2: Summary of studies excluded from PubMed in the systematic review of breast milk caloric density (2005–2025); Supplementary Table S3: Summary of studies excluded from Elicit in the systematic review of breast milk caloric density (2005–2025); Supplementary Table S4: Summary of studies excluded from PubMed in the systematic review of breast milk volume per feeding session (2005–2025); Supplementary Table S5: Summary of studies excluded from Elicit in the systematic review of breast milk volume per feeding session (2005–2025); Supplementary Table S6: Summary of studies excluded from PubMed in the systematic review of breastfeeding frequency (2005–2025); Supplementary Table S7: Summary of studies excluded from Elicit in the systematic review of breastfeeding frequency (2005–2025); Supplementary Table S8: Meta-analysis of caloric density of breast milk (kcal/100 mL) in full-term infants aged 0–1 month, based on systematic review data; Supplementary Table S9: Meta-analysis of caloric density of breast milk (kcal/100 mL) in full-term infants aged 1–3 month, based on systematic review data; Supplementary Table S10: Meta-analysis of caloric density of breast milk (kcal/100 mL) in full-term infants aged 3–6 month, based on systematic review data; Supplementary Table S11: Meta-analysis of caloric density of breast milk (kcal/100 mL) in full-term infants aged 6–9 month, based on systematic review data; Supplementary Table S12: Meta-analysis of caloric density of breast milk (kcal/100 mL) in full-term infants aged 9–12 month, based on systematic review data; Supplementary Table S13: Meta-analysis of caloric density of breast milk (kcal/100 mL) in full-term infants aged 12–18 month, based on systematic review data; Supplementary Table S14: Meta-analysis of caloric density of breast milk (kcal/100 mL) in full-term infants aged 18–24 month, based on systematic review data.

## Abbreviations

The following abbreviations are used in this manuscript:

BM	Breast Milk
CI	Confidence Interval
DTM	Deuterium Dilution Technique
FT-IR	Fourier-Transform Infrared Spectroscopy
GC	Gas Chromatography
HIC	High Caloric Intake
HPLC	High-Performance Liquid Chromatography
IQR	Interquartile Ranges
KCAL	Kilocalories
LCI	Low Caloric Intake
MCI	Medium Caloric Intake
MIRIS	Mid-Infrared Human Milk Analyzer
PERT	Project Evaluation and Review Technique
SD	Standard Deviation
USA	United States of America
WHO	World Health Organization
