# Supplementary material for: Estimating Caloric Intake per Breastfeeding Session in Infants: A Probabilistic Approach
Source: Nutrients. 2025 Sep 30;17(19):3136. doi: 10.3390/nu17193136 (PMC12525735; doi:10.3390/nu17193136)
Supplement: Supplementary file 1 [file nutrients-17-03136-s001.zip › nutrients-3887490-supplementary.pdf]

Appendix-Supplemental Material

Supplemental Table S1. Search strategies used for the systematic reviews. Date of search Aug 2025.

Variable	PubMed search string	Elicit query
**Caloric density**	("breast milk"[Mesh] OR "human milk") AND ("caloric density" OR "energy content" OR "nutrient composition" OR "macronutrient*") AND ("infant"[Mesh] OR infant* OR neonat* OR newborn* OR "0-24 months") AND ("2005/01/01"[Date - Publication] : "2025/12/31"[Date - Publication]) AND "humans"[Filter]	"What are the macronutrient components and caloric density of breast milk in infants aged 0 to 24 months? I'm looking for studies that measure the energy content, including during prolonged lactation beyond 12 months postpartum. Preferably observational or longitudinal studies with direct biochemical analysis. Please include research that provides values by month or age group."
**Volume per feeding**	("Milk, Human"[Mesh] OR "breast milk"[Title/Abstract]) AND ("milk intake"[Title/Abstract] OR "milk volume"[Title/Abstract] OR "milk consumption"[Title/Abstract] OR "breast milk intake"[Title/Abstract]) AND ("feeding"[Title/Abstract] OR "feed"[Title/Abstract] OR "daily intake"[Title/Abstract] OR "per day"[Title/Abstract]) AND ("Infant"[Mesh] OR infant*[Title/Abstract] OR newborn*[Title/Abstract] OR baby[Title/Abstract] OR "0-24 months"[Title/Abstract]) NOT (preterm[Title/Abstract] OR premature[Title/Abstract] OR donor[Title/Abstract] OR banked[Title/Abstract] OR pasteurized[Title/Abstract]) AND ("2005/01/01"[Date - Publication] : "2025/12/31"[Date - Publication])	"What is the average volume of breast milk consumed per feeding session in full-term infants from birth to 24 months of age?"
**Feeding frequency**	("Breast Feeding"[Mesh] OR breastfeeding[tiab]) AND ("feeding frequency"[tiab] OR "number of feeds"[tiab] OR "feeds per day"[tiab] OR "feeding intervals"[tiab]) AND (infant[tiab] OR infants[tiab] OR newborn[tiab] OR baby[tiab]) NOT (preterm[tiab] OR premature[tiab] OR NICU[tiab]) AND ("2005/01/01"[Date - Publication] : "2025/12/31"[Date - Publication])	"How many times per day do full-term infants breastfeed from birth to 24 months of age?"

Supplemental Table S2. Summary of studies excluded from PubMed in the systematic review of breast milk caloric density (2005–2025).

Reason for Exclusion	Title	Citation	First Author
**Narrative/review articles/editorials/ protocols or meta-analyses without primary data**	Artificial intelligence applied to the study of human milk and breastfeeding: a scoping review	Int Breastfeed J. 2024 Dec 6;19(1):79. doi: 10.1186/s13006-024-00686-1.	Agudelo-Pérez S
	Metabolizable Energy Content of Breastmilk Supports Normal Growth in Exclusively Breastfed Icelandic Infants to Age 6 Months	Am J Clin Nutr. 2023 Aug;118(2):468-475. doi: 10.1016/j.ajcnut.2023.06.005. Epub 2023 Jun 13.	Thorisdottir B
	Effect of Different Dietary Patterns on Macronutrient Composition in Human Breast Milk: A Systematic Review and Meta-Analysis	Nutrients. 2023 Jan 17;15(3):485. doi: 10.3390/nu15030485.	Xi Q
	Individualized Fortification Based on Measured Macronutrient Content of Human Milk Improves Growth and Body Composition in Infants Born Less than 33 Weeks: A Mixed-Cohort Study	Nutrients. 2023 Mar 22;15(6):1533. doi: 10.3390/nu15061533.	Cardoso M
	The Impact of Maternal Chronic Inflammatory Conditions on Breast Milk Composition: Possible Influence on Offspring Metabolic Programming	Nutrients. 2025 Jan 22;17(3):387. doi: 10.3390/nu17030387.	Arenas G
	Effect of Epidermal Growth Factor in Human Milk and Maternal Diet on Late-Onset Breast Milk Jaundice: A Case-Control Study in Beijing	Nutrients. 2022 Nov 1;14(21):4587. doi: 10.3390/nu14214587.	Guo Q
	Does the Composition of Breast Milk in the First Week Postpartum Differ Due to Maternal Factors or Neonatal Birth Weight and Percent Fat Body Mass?	Nutrients. 2024 Sep 30;16(19):3310. doi: 10.3390/nu16193310.	Karcz K
	Sex-Specific Effects of Nutritional Supplements for Infants Born Early or Small: An Individual Participant Data Meta-Analysis (ESSENCE IPD-MA) II: Growth	Nutrients. 2022 Jan 17;14(2):392. doi: 10.3390/nu14020392.	Lin L

	Maternal Diet Associated with Oligosaccharide Abundances in Human Milk from Latina Mothers	Nutrients. 2024 Jun 7;16(12):1795. doi: 10.3390/nu16121795.	Mokhtari P
	Development of Visceral and Subcutaneous-Abdominal Adipose Tissue in Breastfed Infants during First Year of Lactation	Nutrients. 2021 Sep 21;13(9):3294. doi: 10.3390/nu13093294.	Gridneva Z
	The Effect of Hyperbaric Storage on the Nutritional Value and Retention of Certain Bioactive Proteins in Human Milk	Nutrients. 2024 May 12;16(10):1455. doi: 10.3390/nu16101455.	Mazur K
	Role of Daily Milk Volume and Period of Lactation in Nutrient Content of Human Milk: Results from a Prospective Study	Nutrients. 2020 Feb 6;12(2):421. doi: 10.3390/nu12020421.	Rigourd V
	Subclinical Mastitis in a European Multicenter Cohort: Prevalence, Impact on Human Milk (HM) Composition, and Association with Infant HM Intake and Growth	Nutrients. 2019 Dec 30;12(1):105. doi: 10.3390/nu12010105.	Samuel TM
	Exclusive or partial breastfeeding and estimated protein intake in infancy: Associations with childhood growth and body composition at 7 years of age in the Odense Child Cohort	J Hum Nutr Diet. 2025 Feb;38(1):e13408. doi: 10.1111/jhn.13408.	Honoré KD
	Validation of Correction Algorithms for Near-IR Analysis of Human Milk in an Independent Sample Set-Effect of Pasteurization	Nutrients. 2016 Feb 26;8(3):119. doi: 10.3390/nu8030119.	Kotrri G
	The potential of a simple egg to improve maternal and child nutrition	Matern Child Nutr. 2018 Oct;14 Suppl 3(Suppl 3):e12678. doi: 10.1111/mcn.12678.	Lutter CK
	A pilot study on nutrients, antimicrobial proteins, and bacteria in commerce-free models for exchanging expressed human milk in the USA	Matern Child Nutr. 2018 Dec;14 Suppl 6(Suppl 6):e12566. doi: 10.1111/mcn.12566.	Perrin MT
	Macronutrients in Human Milk and Early Childhood Growth-Is Protein the Main Driver?	Nutrients. 2024 Oct 16;16(20):3514. doi: 10.3390/nu16203514.	Ma J

	Innovative Techniques of Processing Human Milk to Preserve Key Components	Nutrients. 2019 May 24;11(5):1169. doi: 10.3390/nu11051169.	Wesolowska A
	NIH workshop on human milk composition: summary and visions	Am J Clin Nutr. 2019 Sep 1;110(3):769-779. doi: 10.1093/ajcn/nqz123.	Casavale KO
	A Systematic Review of Collection and Analysis of Human Milk for Macronutrient Composition	J Nutr. 2020 Jun 1;150(6):1652-1670. doi: 10.1093/jn/nxaa059.	Leghi GE
	Macronutrient content of donor milk from a regional human milk bank: variation with donor mother-infant characteristics	Br J Nutr. 2019 Nov 28;122(10):1155-1167. doi: 10.1017/S0007114519002228.	Mills L
	Human Milk Processing and Its Effect on Protein and Leptin Concentrations	Nutrients. 2023 Jan 10;15(2):347. doi: 10.3390/nu15020347.	Binder C
	Lactoferrin in Human Milk of Prolonged Lactation	Nutrients. 2019 Oct 2;11(10):2350. doi: 10.3390/nu11102350.	Czosnykowska-Łukacka M
	Impact of Infant and Maternal Factors on Energy and Macronutrient Composition of Human Milk	Nutrients. 2020 Aug 26;12(9):2591. doi: 10.3390/nu12092591.	Bzikowska-Jura A
	A Systematic Review over the Effect of Early Infant Diet on Neurodevelopment: Insights from Neuroimaging	Nutrients. 2024 May 30;16(11):1703. doi: 10.3390/nu16111703.	Gilbreath D
	Feeding Interventions for Infants with Growth Failure in the First Six Months of Life: A Systematic Review	Nutrients. 2020 Jul 9;12(7):2044. doi: 10.3390/nu12072044.	Rana R
	The impact of maternal obesity on human milk macronutrient composition: A systematic review and meta-analysis	Nutrients. 2020 Mar 27;12(4):934. doi: 10.3390/nu12040934.	Leghi GE
	Nutrition in Necrotizing Enterocolitis and Following Intestinal Resection	Nutrients. 2020 Feb 18;12(2):520. doi: 10.3390/nu12020520.	Ou J
	Dietary Patterns of Breastfeeding Mothers and Human Milk Composition: Data from the Italian MEDIDIET Study	Nutrients. 2021 May 19;13(5):1722. doi: 10.3390/nu13051722.	Bravi F
**No caloric density data or not reported**	Term infant formula macronutrient composition: An update for clinicians	J Pediatr Gastroenterol Nutr. 2025 May;80(5):751-759. doi: 10.1002/jpn3.70002. Epub 2025 Feb 10.	Larson-Nath C

**in the required format (mean ± standard deviation unavailable)**	Relationship Between Maternal Age and Macronutrient Content of Colostrum	J Hum Lact. 2024 May;40(2):286-295. doi: 10.1177/08903344241233500. Epub 2024 Feb 27.	Hochman VGA
	Processing Human Milk to Increase Nutrient Density for Preterm Infants	J Hum Lact. 2023 May;39(2):333-342. doi: 10.1177/08903344211056933. Epub 2021 Nov 15.	Ulus HZ
	Changes in Mature Human Milk Macronutrient Composition over 45 years in an Urban Population in Indonesia	J Hum Lact. 2023 Nov;39(4):648-655. doi: 10.1177/08903344231195326. Epub 2023 Sep 15.	Nurani N
	Donor Human Milk Fat Content Is Associated with Maternal Body Mass Index	Breastfeed Med. 2025 Feb;20(2):126-132. doi: 10.1089/bfm.2024.0028. Epub 2024 Nov 26.	de Castro LS
	Optimal Distribution and Utilization of Donated Human Breast Milk	J Hum Lact. 2016 Nov;32(4):730-734. doi: 10.1177/0890334416653738. Epub 2016 Jul 10.	Simpson JH
	Nutrients and Bioactive Components of Human Milk After One Year of Lactation: Implication for Human Milk Banks	J Pediatr Gastroenterol Nutr. 2022 Feb 1;74(2):284-291. doi: 10.1097/MPG.0000000000003298.	Sinkiewicz-Darol E
	Human milk composition differs by maternal BMI in the first 9 months postpartum	Am J Clin Nutr. 2020 Sep 1;112(3):548-557. doi: 10.1093/ajcn/nqaa098.	Sims CR
	Longitudinal human milk macronutrients, body composition and infant appetite during early life	Clin Nutr. 2021 May;40(5):3401-3408. doi: 10.1016/j.clnu.2020.11.024. Epub 2020 Nov 24.	de Fluiter KS
	Macronutrient composition of term and preterm human milk of different socio economic groups	Prostaglandins Leukot Essent Fatty Acids. 2023 May;192:102571. doi: 10.1016/j.plefa.2023.102571. Epub 2023 Mar 30.	K B C
	Maternal Lifestyle Factors Affecting Breast Milk Composition and Infant Health: A Systematic Review	Nutrients. 2024 Dec 27;17(1):62. doi: 10.3390/nu17010062.	Favara G
	Nutritional composition of breast milk in Chinese women: a systematic review	Asia Pac J Clin Nutr. 2018;27(3):491-502. doi: 10.6133/apjcn.042017.13.	Yang T
	The impact of human breast milk components on the infant metabolism	PLoS One. 2018 Jun 1;13(6):e0197713. doi: 10.1371/journal.pone.0197713. eCollection 2018.	Hellmuth C

	The association of pre-pregnancy BMI on leptin, ghrelin, adiponectin and insulin-like growth factor-1 in breast milk: a case-control study	Br J Nutr. 2022 Jun 14;127(11):1675-1681. doi: 10.1017/S0007114521002932. Epub 2021 Aug 5.	Tekin Guler T
	Is Frozen Human Milk That Is Refused by Mother's Own Infant Suitable for Human Milk Bank Donation?	Breastfeed Med. 2019 May;14(4):271-275. doi: 10.1089/bfm.2018.0193. Epub 2019 Feb 21.	Pitino MA
	The Effect of Cannabis Consumption During Lactation on the Macronutrient Concentrations in Breast Milk	Breastfeed Med. 2025 Jan;20(1):33-41. doi: 10.1089/bfm.2024.0083. Epub 2024 Nov 12.	Narayanan P
	Experience of Induced Lactation in a Transgender Woman: Analysis of Human Milk and a Suggested Protocol	Breastfeed Med. 2023 Nov;18(11):888-893. doi: 10.1089/bfm.2023.0197. Epub 2023 Nov 1.	Delgado D
	Dietary Intake of Chinese Lactating Women Is Associated with the Fatty Acid Profile of Their Milk	Ann Nutr Metab. 2022;78(1):33-45. doi: 10.1159/000520515. Epub 2021 Dec 2.	Montez de Sousa ÍR
	Effect of Donated Premature Milk in the Prevention of Bronchopulmonary Dysplasia	Nutrients. 2024 Mar 15;16(6):859. doi: 10.3390/nu16060859.	Merino-Hernández A
	Effect of Pooling Practices and Time Postpartum of Milk Donations on the Energy, Macronutrient, and Zinc Concentrations of Resultant Donor Human Milk Pools	J Pediatr. 2019 Nov;214:54-59. doi: 10.1016/j.jpeds.2019.07.042. Epub 2019 Sep 23.	Young BE
	Dietary patterns affect maternal macronutrient intake levels and the fatty acid profile of breast milk in lactating Chinese mothers	Nutrition. 2019 Feb;58:83-88. doi: 10.1016/j.nut.2018.06.009. Epub 2018 Jul 11.	Tian HM
	Nutritional profile of newborns with microcephaly and factors associated with worse outcomes	Clinics (Sao Paulo). 2019 Oct 21;74:e798. doi: 10.6061/clinics/2019/e798. eCollection 2019.	Dos Santos SFM
	Proactive Use of a Human Milk Fat Modular in the Neonatal Intensive Care Unit: A Standardized Feeding Protocol	Nutrients. 2024 Apr 18;16(8):1206. doi: 10.3390/nu16081206.	Salley A
	Tandem Breastfeeding: A Descriptive Analysis of the Nutritional Value of Milk	Nutrients. 2021 Jan 19;13(1):277. doi: 10.3390/nu13010277.	Sinkiewicz-Darol E

	When Feeding a Younger and Older Child		
	Comparison of the Effect of Three Different Fortification Methods on Growth of Very Low Birth Weight Infants	Breastfeed Med. 2019 Jan/Feb;14(1):63-68. doi: 10.1089/bfm.2018.0093. Epub 2018 Nov 28.	Kadıoğlu Şimşek G
	The Practicality of Preparing Skim Breast Milk at Home for Treatment of Infants Requiring Low Fat Diets	Breastfeed Med. 2024 Mar;19(3):217-222. doi: 10.1089/bfm.2023.0299.	Huang Y
	Composition and Variation of Macronutrients, Immune Proteins, and Human Milk Oligosaccharides in Human Milk From Nonprofit and Commercial Milk Banks	J Hum Lact. 2018 Feb;34(1):120-129. doi: 10.1177/0890334417710635. Epub 2017 Jun 14.	Meredith-Dennis L
	Immediate effect of food intake by the nursing mother on the macronutrient content of colostrum	J Pediatr (Rio J). 2025 Jul-Aug;101(4):584-589. doi: 10.1016/j.jped.2025.03.004. Epub 2025 Apr 3.	Nascimento RCFA
	Multiple Approaches Detect the Presence of Fungi in Human Breastmilk Samples from Healthy Mothers	Sci Rep. 2017 Oct 12;7(1):13016. doi: 10.1038/s41598-017-13270-x.	Boix-Amorós A
	Associations between breast milk intake volume, macronutrient intake and infant growth in a longitudinal birth cohort: the Cambridge Baby Growth and Breastfeeding Study (CBGS-BF)	Br J Nutr. 2023 Jul 14;130(1):56-64. doi: 10.1017/S0007114522003178. Epub 2022 Oct 19.	Olga L
	Gestational Diabetes Mellitus, Human Milk Composition, and Infant Growth	Breastfeed Med. 2023 Jan;18(1):14-22. doi: 10.1089/bfm.2022.0085. Epub 2022 Nov 21.	Dugas C
	Macronutrient content of pooled donor human milk before and after Holder pasteurization	BMC Pediatr. 2019 Feb 12;19(1):58. doi: 10.1186/s12887-019-1427-5.	Piemontese P
	Effect of Ramadan Fasting on Breast Milk	Breastfeed Med. 2023 Aug;18(8):596-601. doi: 10.1089/bfm.2023.0144.	Başıbüyük M
	Human breast milk-based nutritherapy: A blueprint for pediatric healthcare	J Food Drug Anal. 2021 Jun 15;29(2):203-213. doi: 10.38212/2224-6614.3352.	Shende P
	Human milk composition and infant anthropometrics: overview of a systematic review with clinical and research implications	Int Breastfeed J. 2024 Jun 28;19(1):45. doi: 10.1186/s13006-024-00652-x.	Azad MB

	Breast milk nutrient content and infancy growth	Acta Paediatr. 2016 Jun;105(6):641-7. doi: 10.1111/apa.13362. Epub 2016 Apr 6.	Prentice P
	25 Years of Research in Human Lactation: From Discovery to Translation	Nutrients. 2021 Aug 31;13(9):3071. doi: 10.3390/nu13093071.	Geddes DT
	Pooling Strategies to Modify Macronutrient Content of Pasteurized Donor Human Milk	Breastfeed Med. 2023 May;18(5):370-376. doi: 10.1089/bfm.2023.0043. Epub 2023 Apr 25.	Tabasso C
**Non-quantitative data**	Assessment of the Composition of Breastmilk Substitutes, Commercial Complementary Foods, and Commercial Snack Products Commonly Fed to Infant and Young Children in Lebanon: A Call to Action	Nutrients. 2023 Feb 27;15(5):1200. doi: 10.3390/nu15051200.	Hoteit M
	Analysis of the Storage Methods for Raw Human Milk from Mothers with Infants Admitted to a Neonatal Intensive Care Unit, According to Brazilian Regulations	J Hum Lact. 2016 Aug;32(3):446-54. doi: 10.1177/0890334416647710. Epub 2016 May 10.	Grazziotin MC
	Buffered or impaired: Maternal anemia, inflammation and breast milk macronutrients in northern Kenya	Am J Phys Anthropol. 2019 Feb;168(2):329-339. doi: 10.1002/ajpa.23752. Epub 2018 Dec 21.	Fujita M
	Cannabis use during lactation may alter the composition of human breast milk	Pediatr Res. 2023 Jun;93(7):1959-1968. doi: 10.1038/s41390-022-02315-1. Epub 2022 Oct 4.	Josan C
**Methodological/biochemical studies without applicable caloric density values**	Macronutrient Analysis of Target-Pooled Donor Breast Milk and Corresponding Growth in Very Low Birth Weight Infants	Nutrients. 2019 Aug 13;11(8):1884. doi: 10.3390/nu11081884.	Fu TT
	Macronutrient Intake from Human Milk, Infant Growth, and Body Composition at Term Equivalent Age: A Longitudinal Study of Hospitalized Very Preterm Infants	Nutrients. 2020 Jul 28;12(8):2249. doi: 10.3390/nu12082249.	Belfort M
	Reduction in Maternal Energy Intake during Lactation Decreased Maternal Body Weight and Concentrations of Leptin, Insulin and Adiponectin in Human Milk	Nutrients. 2021 May 31;13(6):1892. doi: 10.3390/nu13061892.	Leghi GE

	without Affecting Milk Production, Milk Macronutrient Composition or Infant Growth		
	Sex-Specific Effects of Nutritional Supplements for Infants Born Early or Small: An Individual Participant Data Meta-Analysis (ESSENCE IPD-MA) I-Cognitive Function and Metabolic Risk	Nutrients. 2022 Jan 18;14(3):418. doi: 10.3390/nu14030418.	Lin L
	Impact of Maternal Anxiety on Human Milk Macronutrients Content: A Prospective Observational Study	Breastfeed Med. 2020 Sep;15(9):572-575. doi: 10.1089/bfm.2020.0034. Epub 2020 Jun 26.	Palnizky Soffer G
	The Effect of Prolonged Freezing and Holder Pasteurization on the Macronutrient and Bioactive Protein Compositions of Human Milk	Breastfeed Med. 2020 Sep;15(9):583-588. doi: 10.1089/bfm.2020.0219. Epub 2020 Aug 26.	Paulaviciene IJ
	Effect of Human Milk Appetite Hormones, Macronutrients, and Infant Characteristics on Gastric Emptying and Breastfeeding Patterns of Term Fully Breastfed Infants	Nutrients. 2016 Dec 28;9(1):15. doi: 10.3390/nu9010015.	Gridneva Z
	Infrared analyzers for breast milk analysis: fat levels can influence the accuracy of protein measurements	Clin Chem Lab Med. 2017 Oct 26;55(12):1931-1935. doi: 10.1515/cclm-2016-1042.	Kwan C
	Proteomic analysis of milk fat globule membrane proteins in mature human milk of women with and without gestational diabetes mellitus	Food Chem. 2024 Jul 1;445:138691. doi: 10.1016/j.foodchem.2024.138691. Epub 2024 Feb 8.	Yao D
	Lipid-Based Nutrient Supplements Providing Approximately the Recommended Daily Intake of Vitamin A Do Not Increase Breast Milk Retinol Concentrations among Ghanaian Women	J Nutr. 2016 Feb;146(2):335-42. doi: 10.3945/jn.115.217786. Epub 2016 Jan 6.	Klevor MK
	Association between fat-soluble vitamins in breast milk and neonatal gut microbiome in Tibetan mother-infant dyads during the first month postnatal	Food Res Int. 2025 Jul;212:116350. doi: 10.1016/j.foodres.2025.116350. Epub 2025 Apr 28.	Zhang X

	Dynamic Changes in Antibodies and Proteome in Breast Milk of Mothers Infected with Wild-Type SARS-CoV-2 and Omicron: A Longitudinal Study	Nutrients. 2025 Apr 21;17(8):1396. doi: 10.3390/nu17081396.	Guo Y
	Effect of hepatitis B virus infection on the nutrient composition of human breast milk: A prospective cohort study	Food Chem. 2025 Feb 15;465(Pt 2):141947. doi: 10.1016/j.foodchem.2024.141947. Epub 2024 Nov 13.	Zhang P
	Associations between Maternal Body Composition and Appetite Hormones and Macronutrients in Human Milk	Nutrients. 2017 Mar 9;9(3):252. doi: 10.3390/nu9030252.	Kugananthan S
	Sex-Specific Human Milk Composition: The Role of Infant Sex in Determining Early Life Nutrition	Nutrients. 2018 Sep 1;10(9):1194. doi: 10.3390/nu10091194.	Galante L
	Tandem Breastfeeding and Human Milk Macronutrients: A Prospective Observational Study	J Hum Lact. 2021 Nov;37(4):723-729. doi: 10.1177/08903344211003827. Epub 2021 Mar 21.	Rosenberg G
	Nesfatin-1 in Human Milk and Its Association with Infant Anthropometry	Nutrients. 2022 Dec 30;15(1):176. doi: 10.3390/nu15010176.	Honoré KD
	Macronutrient Composition of Donated Human Milk in a New Zealand Population	J Hum Lact. 2021 Feb;37(1):114-121. doi: 10.1177/0890334420963666. Epub 2020 Oct 8.	Lamb RL
**No stratification by infant age group**	Correlation between human milk composition and maternal nutritional status	Rocz Panstw Zakl Hig. 2018;69(4):363-367. doi: 10.32394/rpzh.2018.0041.	Bzikowska A
	Dietary patterns and their association with breast milk macronutrient composition among lactating women	Int Breastfeed J. 2020 Jun 5;15(1):52. doi: 10.1186/s13006-020-00293-w.	Huang Z
	Circadian changes in the composition of human milk macronutrients depending on pregnancy duration: a cross-sectional study	Int Breastfeed J. 2020 May 25;15(1):49. doi: 10.1186/s13006-020-00291-y.	Paulaviciene IJ
	Maternal and Infant Predictors of Human Milk Macronutrient and Energy Concentrations in Rural Bangladesh: An Observational Cohort Study	J Nutr. 2025 Apr;155(4):1151-1159. doi: 10.1016/j.tjnut.2024.12.027. Epub 2024 Dec 31.	North K
	From Mother-Fetus Dyad to Mother-Milk-Infant Triad: Sex	Nutrients. 2025 Apr 23;17(9):1422. doi: 10.3390/nu17091422.	Lithoxopoulou M

	Differences in Macronutrient Composition of Breast Milk		
	Association of maternal breast milk and serum levels of macronutrients, hormones, and maternal body composition with infant's body weight	Eur J Clin Nutr. 2018 Mar;72(3):394-400. doi: 10.1038/s41430-017-0022-9. Epub 2017 Nov 23.	Khodabakhshi A
	Relationship Between Birthweight for Gestational Age and Colostrum Macronutrient Composition: A Comparative Analysis in Small, Appropriate, and Large for Gestational Age Infants	J Hum Lact. 2025 Aug;41(3):371-378. doi: 10.1177/08903344251342561. Epub 2025 Jun 29.	Arayici S
	Appetite-regulating hormone trajectories and relationships with fat mass development in term-born infants during the first 6 months of life	Eur J Nutr. 2021 Oct;60(7):3717-3725. doi: 10.1007/s00394-021-02533-z. Epub 2021 Mar 25.	de Fluiter KS
	Associations between Maternal Nutrition and the Concentrations of Human Milk Oligosaccharides in a Cohort of Healthy Australian Lactating Women	Nutrients. 2023 Apr 26;15(9):2093. doi: 10.3390/nu15092093.	Biddulph C
	Extensive Study of Breast Milk and Infant Growth: Protocol of the Cambridge Baby Growth and Breastfeeding Study (CBGS-BF)	Nutrients. 2021 Aug 21;13(8):2879. doi: 10.3390/nu13082879.	Olga L
	Factors That May Affect Breast Milk Macronutrient and Energy Content: A Critical Review	Nutrients. 2025 Jul 30;17(15):2503. doi: 10.3390/nu17152503.	Rocha-Pinto I
	Circadian Variation in Human Milk Hormones and Macronutrients	Nutrients. 2023 Aug 25;15(17):3729. doi: 10.3390/nu15173729.	Suwaydi MA
	Maternal nutritional status and milk volume and composition in India: an observational study	Am J Clin Nutr. 2023 Apr;117(4):830-837. doi: 10.1016/j.ajcnut.2023.02.002. Epub 2023 Feb 10.	Young MF
**Non-applicable population**	Comparison Between Antenatal and Postnatal Colostrum From Women With and Without Type 1 Diabetes	J Hum Lact. 2025 May;41(2):254-262. doi: 10.1177/08903344251318285. Epub 2025 Mar 12.	Goldberg A

	Gestational Hypertension and Human Breast Milk Composition in Correlation with the Assessment of Fetal Growth-A Pilot Study	Nutrients. 2023 May 21;15(10):2404. doi: 10.3390/nu15102404.	Sokołowska EM
	Immunological Factors and Macronutrient Content in Human Milk From Women With Subclinical Mastitis	J Hum Lact. 2025 Feb;41(1):26-33. doi: 10.1177/08903344241297585. Epub 2024 Dec 20.	Ito M
	Determinants of bone mineral density in healthy term-born children at age 6 months and 3 years	Clin Nutr. 2025 Aug;51:304-313. doi: 10.1016/j.clnu.2025.07.001. Epub 2025 Jul 3.	Dorrepaal DJ

Supplemental Table S3. Summary of studies excluded from Elicit in the systematic review of breast milk caloric density (2005–2025).

Reason for Exclusion	Title	Citation	First Author
**No caloric density data or not reported in the required format (mean ± standard deviation unavailable)**	Longitudinal human milk macronutrients, body composition and infant appetite during early life	Clin Nutr. 2021 May;40(5):3401-3408. doi: 10.1016/j.clnu.2020.11.024. Epub 2020 Nov 24.	de Fluiter KS
**Narrative/review articles/editorials/ protocols or meta-analyses without primary data**	Is the macronutrient intake of formula-fed infants greater than breast-fed infants in early infancy?	Nutr Metab. 2012;2012:891201. doi:10.1155/2012/891201	Hester SN

Supplemental Table S4. Summary of studies excluded from PubMed in the systematic review of breast milk volume per feeding session (2005–2025).

Reason for Exclusion	Title	Citation	First Author
**Narrative/review articles/editorials/protocols or meta-analyses without primary data**	The Volume of Breast Milk Intake in Infants and Young Children: A Systematic Review and Meta-Analysis	Breastfeed Med. 2023 Mar;18(3):188-197. doi: 10.1089/bfm.2022.0281. Epub 2023 Feb 10.	Rios-Leyvraz M
	Human Milk Nutrient Composition Data is Critically Lacking in the United States and Canada: Results from a Systematic Scoping Review of 2017-2022	Adv Nutr. 2023 Nov;14(6):1617-1632. doi: 10.1016/j.advnut.2023.09.007. Epub 2023 Sep 25.	Mohr AE
**Non-applicable population**	Breastfeeding frequency, milk volume, and duration in mother-infant dyads with persistent nipple pain	Breastfeed Med. 2012 Aug;7:275-81. doi: 10.1089/bfm.2011.0117. Epub 2012 Mar 16.	McClellan HL
	Breastfeeding and smoking: short-term effects on infant feeding and sleep	Pediatrics. 2007 Sep;120(3):497-502. doi: 10.1542/peds.2007-0488.	Mennella JA
	Breast-milk intake of 9-10-mo-old rural infants given a ready-to-use complementary food in South Kivu, Democratic Republic of Congo	Am J Clin Nutr. 2011 Jun;93(6):1300-4. doi: 10.3945/ajcn.110.006544. Epub 2011 Mar 30.	Owino VO
	Greater household food insecurity is associated with lower breast milk intake among infants in western Kenya	Matern Child Nutr. 2019 Oct;15(4):e12862. doi: 10.1111/mcn.12862. Epub 2019 Jul 29.	Miller JD
	Effects of varied energy density of complementary foods on breast-milk intakes and total energy consumption by healthy, breastfed Bangladeshi children	Am J Clin Nutr. 2006 Apr;83(4):851-8. doi: 10.1093/ajcn/83.4.851.	Islam MM
	Use of stable-isotope techniques to validate infant feeding practices reported by Bangladeshi women receiving breastfeeding counseling	Am J Clin Nutr. 2007 Apr;85(4):1075-82. doi: 10.1093/ajcn/85.4.1075.	Moore SE
	Breastfeeding practices of Cameroonian mothers determined by dietary recall since birth and the dose-to-the-mother	Matern Child Nutr. 2012 Jul;8(3):330-9. doi: 10.1111/j.1740-8709.2011.00293.x. Epub 2011 Mar 22.	Medoua GN

	deuterium-oxide turnover technique		
	Breast milk intake is not reduced more by the introduction of energy dense complementary food than by typical infant porridge	J Nutr. 2007 Jul;137(7):1828-33. doi: 10.1093/jn/137.7.1828.	Galpin L
	Reliability and validity of swallows as a measure of breast milk intake in the first days of life	J Hum Lact. 2012 Nov;28(4):483-9. doi: 10.1177/0890334412451364. Epub 2012 Jul 21.	Côté-Arsenault D
	Effects of energy density and feeding frequency of complementary foods on total daily energy intakes and consumption of breast milk by healthy breastfed Bangladeshi children	Am J Clin Nutr. 2008 Jul;88(1):84-94. doi: 10.1093/ajcn/88.1.84.	Islam MM
	Maternal Body Composition and Its Relationship to Infant Breast Milk Intake in Rural Pakistan	Food Nutr Sci. 2011 Nov;2(9):932-937. doi: 10.4236/fns.2011.29127.	Nazlee N
	Assessment of body composition and breast milk volume in lactating mothers in pastoral communities in Pokot, Kenya, using deuterium oxide	Ann Nutr Metab. 2005 Mar-Apr;49(2):110-7. doi: 10.1159/000084744. Epub 2005 Mar 29.	Ettyang GA
**No milk volume data**	Human milk feeding practices and serum immune profiles of one-year-old infants in the CHILD birth cohort study	Am J Clin Nutr. 2025 Jan;121(1):60-73. doi: 10.1016/j.ajcnut.2024.10.021. Epub 2024 Oct 30.	Ames SR
	Breast milk sodium concentration, sodium intake and weight loss in breast-feeding newborn infants	Br J Nutr. 2007 Feb;97(2):344-8. doi: 10.1017/S0007114507280572.	Manganaro R
**No milk volume data per feeding**	Impact of maternal diet on human milk composition and neurological development of infants	Am J Clin Nutr. 2014 Mar;99(3):734S-41S. doi: 10.3945/ajcn.113.072595. Epub 2014 Feb 5.	Innis SM
	Breast milk composition and infant nutrient intakes during the first 12 months of life	Eur J Clin Nutr. 2016 Feb;70(2):250-6. doi: 10.1038/ejcn.2015.162. Epub 2015 Sep 30.	Grote V
	Associations between breast milk intake volume, macronutrient intake and	Br J Nutr. 2023 Jul 14;130(1):56-64. doi:	Olga L

	infant growth in a longitudinal birth cohort: the Cambridge Baby Growth and Breastfeeding Study (CBGS-BF)	10.1017/S0007114522003178. Epub 2022 Oct 19.	
	Human Milk Casein and Whey Protein and Infant Body Composition over the First 12 Months of Lactation	Nutrients. 2018 Sep 19;10(9):1332. doi: 10.3390/nu10091332.	Gridneva Z
	Carbohydrates in Human Milk and Body Composition of Term Infants during the First 12 Months of Lactation	Nutrients. 2019 Jun 28;11(7):1472. doi: 10.3390/nu11071472.	Gridneva Z
	Adequacy of milk intake during exclusive breastfeeding: a longitudinal study	Pediatrics. 2011 Oct;128(4):e907-14. doi: 10.1542/peds.2011-0914. Epub 2011 Sep 19.	Nielsen SB
**No stratification by infant age**	Volume and frequency of breastfeedings and fat content of breast milk throughout the day	Pediatrics. 2006 Mar;117(3):e387-95. doi: 10.1542/peds.2005-1417.	Kent JC

Supplemental Table S5. Summary of studies excluded from Elicit in the systematic review of breast milk volume per feeding session (2005–2025).

Reason for Exclusion	Title	Citation	First Author
**No milk volume data**	Breast milk composition and infant nutrient intakes during the first 12 months of life	Eur J Clin Nutr. 2016 Feb;70(2):250-6. doi: 10.1038/ejcn.2015.162. Epub 2015 Sep 30.	Grote V
	Associations between breast milk intake volume, macronutrient intake and infant growth in a longitudinal birth cohort: the Cambridge Baby Growth and Breastfeeding Study (CBGS-BF)	Br J Nutr. 2023 Jul 14;130(1):56-64. doi: 10.1017/S0007114522003178. Epub 2022 Oct 19.	Olga L
**No stratification by infant age**	Volume and frequency of breastfeedings and fat content of breast milk throughout the day	Pediatrics. 2006 Mar;117(3):e387-95. doi: 10.1542/peds.2005-1417.	Kent JC
	Longitudinal changes in breastfeeding patterns from 1 to 6 months of lactation	Breastfeed Med. 2013;8(4):401–7. doi:10.1089/bfm.2012.0141	Kent JC

Supplemental Table S6. Summary of studies excluded from PubMed in the systematic review of breastfeeding frequency (2005–2025).

Reason for Exclusion	Title	Citation	First Author
**No valid feeding frequency data**	Association of Maternal Confidence and Breastfeeding Practices in Hispanic Women Compared to Non-Hispanic White Women	Glob Pediatr Health. 2021 Dec 22;8:2333794X211062439. doi: 10.1177/2333794X211062439. eCollection 2021.	Hoyt-Austin A
	Indicators of 'good' feeding, breastfeeding latch, and feeding experiences among healthy women with healthy infants: A qualitative pathway analysis using Grounded Theory	Women Birth. 2021 Jul;34(4):e357-e367. doi: 10.1016/j.wombi.2020.08.004. Epub 2020 Sep 1.	Davie P
	Eating Frequency in European Children from 1 to 96 Months of Age: Results of the Childhood Obesity Project Study	Nutrients. 2023 Feb 16;15(4):984. doi: 10.3390/nu15040984.	Jaeger V
**Non-applicable population**	Infant and child feeding index reflects feeding practices, nutritional status of urban slum children	BMC Pediatr. 2014 Nov 30;14:290. doi: 10.1186/s12887-014-0290-7.	Lohia N
	Daily meal frequency and its associated factors among children aged 6-23 months in Ethiopia: a Bayesian hierarchical Poisson model	Front Public Health. 2025 Jul 18;13:1563392. doi: 10.3389/fpubh.2025.1563392. eCollection 2025.	Asgedom DK
	Short-term effect of complementary feeding frequency on total ad libitum consumption in 6- to 10-month-old breast fed Indian infants	J Pediatr Gastroenterol Nutr. 2005 Oct;41(4):422-5. doi: 10.1097/01.mpg.0000178443.18041.98.	Singh PK

Supplemental Table S7. Summary of studies excluded from Elicit in the systematic review of breastfeeding frequency (2005–2025).

Reason for Exclusion	Title	Citation	First Author
**No valid feeding frequency data**	Adequacy of Milk Intake During Exclusive Breastfeeding: A Longitudinal Study	Pediatrics. 2011 Oct;128(4):e907-14. doi: 10.1542/peds.2011-0914. Epub 2011 Sep 19. PMID: 21930538.	Nielsen SB
	Feeding Patterns of Healthy Term Newborns in the First 5 Days-The Glucose in Well Babies Study (GLOW)	J Hum Lact. 2022 Nov;38(4):661-669. doi: 10.1177/08903344221087605. Epub 2022 Apr 6. PMID: 35383475.	Harris DL
**Non-applicable population**	Longitudinal study of the frequency and duration of breastfeeding in rural Bangladeshi women	Am J Hum Biol. 2006;18(5):630-638. doi: 10.1002/ajhb.20533.	Ghosh R

Supplemental Table S8. Meta-analysis of caloric density of breast milk (kcal/100 mL) in full-term infants aged 0–1 month, based on systematic review data.

Age group (month)	Study	Individual value (kcal/100mL) (Mean ± SD)	Total	Weight	Mean IV, Random, 95%, CI	Forest Plot of Mean Caloric Density of Human Milk With 95% CI
0-1	Fischer Fumeaux et al., 2019	55.22 ± 7.08	8	7.3%	55.22 [50.31; 60.13]	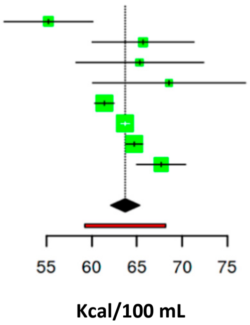
	Fischer Fumeaux et al., 2019	65.68 ± 7.60	7	6.0%	65.68 [60.05; 71.31]	
	Fischer Fumeaux et al., 2019	65.32 ± 10.18	8	4.2%	65.32 [58.27; 72.37]	
	Fischer Fumeaux et al., 2019	68.55 ± 12.20	8	3.1%	68.55 [60.10; 77.00]	
	Chang et al., 2015	61.4 ± 8.5	246	21.1%	61.40 [60.34; 62.46]	
	Chang et al., 2015	63.7 ± 8.0	894	22.7%	63.70 [63.18; 64.22]	
	Chang et al., 2015	64.7 ± 8.3	303	21.5%	64.70 [63.77; 65.63]	
	Saarela et al., 2005	67.68 ± 9.58	48	14.0%	67.68 [64.97; 70.39]	
**Total (95% CI)**			**1522**	100.0%	**63.70 [62.10; 65.31]**	
Heterogeneity: Tau^2=2.8990; Chi^2=43.92, df= 7 (P < 0.0001); I^2=84.1%						

Forest plot showing individual study estimates and pooled mean of breast milk caloric density (kcal/100 mL) in full-term infants aged 0–1 month. Each green square represents the mean and 95% CI of a subgroup, with square size reflecting its weight in the meta-analysis. The black diamond represents the pooled estimate (random-effects model). The squares are ordered as in the table. Some studies contributed multiple data points due to different time points.

Abbreviations: SD: standard deviation; CI: confidence interval; IV: inverse variance.

Source: Based on data from Fischer Fumeaux et al., 2019; Chang et al., 2015; Saarela et al., 2005.

Supplemental Table S9. Meta-analysis of caloric density of breast milk (kcal/100 mL) in full-term infants aged 1–3 month, based on systematic review data.

Age group (month)	Study	Individual value (kcal/100mL) (Mean ± SD)	Total	Weight	Mean IV, Random, 95%, CI	Forest Plot of Mean Caloric Density of Human Milk With 95% CI
1-3	Fischer Fumeaux et al., 2019	58.55 ± 9.18	7	6.9%	58.55 [51.75; 65.35]	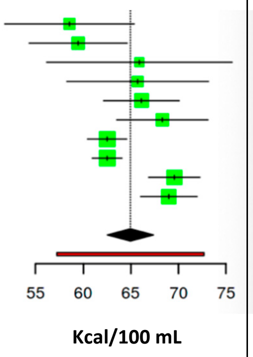
	Fischer Fumeaux et al., 2019	59.46 ± 7.43	8	9.0%	59.46 [54.31; 64.61]	
	Fischer Fumeaux et al., 2019	65.89 ± 14.06	8	4.4%	65.89 [56.15; 75.63]	
	Fischer Fumeaux et al., 2019	65.71 ± 10.74	8	6.3%	65.71 [58.27; 73.15]	
	Grote et al. 2016	66.1 ± 11.1	30	10.8%	66.10 [62.13; 70.07]	
	Grote et al., 2016	68.3 ± 13.4	30	9.5%	68.30 [63.50; 73.10]	
	Chang et al., 2015	62.5 ± 12.3	137	13.7%	62.50 [60.44; 64.56]	
	Chang et al., 2015	62.5 ± 11.9	222	14.3%	62.50 [60.93; 64.07]	
	Saarela et al., 2005	69.56 ± 9.31	46	12.8%	69.56 [66.87; 72.25]	
	Saarela et al., 2005	68.99 ± 9.94	43	12.4%	68.99 [66.02; 71.96]	
**Total (95% CI)**			**539**	100.0%	**64.95 [62.52; 67.38]**	
Heterogeneity: Tau^2=10.1309; Chi^2=42.06, df= 9 (P =0.0010); I^2=78.6%						

Forest plot showing individual study estimates and pooled mean of breast milk caloric density (kcal/100 mL) in full-term infants aged 1–3 months. Each green square represents the mean and 95% CI of a subgroup, with square size reflecting its weight in the meta-analysis. The black diamond represents the pooled estimate (random-effects model). The squares are ordered as in the table. Some studies contributed multiple data points due to different time points.

Abbreviations: SD: standard deviation; CI: confidence interval; IV: inverse variance.

Source: Based on data from Fischer Fumeaux et al., 2019; Grote et al., 2016; Chang et al., 2015; Saarela et al., 2005.

Supplemental Table S10. Meta-analysis of caloric density of breast milk (kcal/100 mL) in full-term infants aged 3–6 month, based on systematic review data.

Age group (month)	Study	Individual value (kcal/100mL) (Mean ± SD)	Total	Weight	Mean IV, Random, 95%, CI	Forest Plot of Mean Caloric Density of Human Milk With 95% CI
3-6	Grote et al., 2016	63.0 ± 10.5	30	10.0%	63.00 [59.24; 66.76]	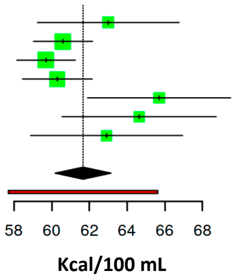
	Chang et al., 2015	60.6 ± 12.1	230	21.3%	60.60 [59.04; 62.16]	
	Chang et al., 2015	59.7 ± 12.1	235	21.4%	59.70 [58.15; 61.25]	
	Chang et al., 2015	60.3 ± 11.5	149	19.5%	60.30 [58.46; 62.15]	
	Saarela et al., 2005	65.70 ± 11.78	37	9.9%	65.70 [61.89; 69.50]	
	Saarela et al., 2005	64.64 ± 12.17	34	8.9%	64.64 [60.58; 68.71]	
	Saarela et al., 2005	62.91 ±10.28	25	9.1%	62.91 [58.86; 66.94]	
**Total (95% CI)**			**740**	100.0%	**61.66 [60.18; 63.15]**	
Heterogeneity: Tau^2=2.0554; Chi^2=14.37, df= 6 (P 0.0258); I^2=58.2%						

Forest plot showing individual study estimates and pooled mean of breast milk caloric density (kcal/100 mL) in full-term infants aged 3–6 months. Each green square represents the mean and 95% CI of a subgroup, with square size reflecting its weight in the meta-analysis. The black diamond represents the pooled estimate (random-effects model). The squares are ordered as in the table. Some studies contributed multiple data points due to different time points.
Abbreviations: SD: standard deviation; CI: confidence interval; IV: inverse variance.
Source: Based on data from Grote et al., 2016; Chang et al., 2015; Saarela et al., 2005.

Supplemental Table S11. Meta-analysis of caloric density of breast milk (kcal/100 mL) in full-term infants aged 6–9 month, based on systematic review data.

Age group (month)	Study	Individual value (kcal/100mL) (Mean ± SD)	Total	Weight	Mean IV, Random, 95%, CI	Forest Plot of Mean Caloric Density of Human Milk With 95% CI
6-9	Grote et al., 2016	62.4 ± 13.3	30	10.6%	62.40 [57.64; 67.16]	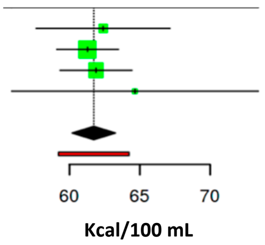
	Chang et al., 2015	61.3 ± 12.0	115	49.7%	61.30 [59.11; 63.49]	
	Chang et al., 2015	61.9 ± 13.1	101	36.6%	61.90 [59.35; 64.45]	
	Saarela et al., 2005	64.67 ± 10.83	20	3.1%	64.67 [55.90; 73.44]	
**Total (95% CI)**			**266**	100.0%	**61.74 [60.19; 63.29]**	
Heterogeneity: Tau^2=0; Chi^2=0,67, df= 3 (P 0.8796); I^2=0.0%						

Forest plot showing individual study estimates and pooled mean of breast milk caloric density (kcal/100 mL) in full-term infants aged 6–9 months. Each green square represents the mean and 95% CI of a subgroup, with square size reflecting its weight in the meta-analysis. The black diamond represents the pooled estimate (random-effects model). The squares are ordered as in the table. Some studies contributed multiple data points due to different time points.
Abbreviations: SD: standard deviation; CI: confidence interval; IV: inverse variance.
Source: Based on data from Grote et al., 2016; Chang et al., 2015; Saarela et al., 2005.

Supplemental Table S12. Meta-analysis of caloric density of breast milk (kcal/100 mL) in full-term infants aged 9–12 month, based on systematic review data.

Age group (month)	Study	Individual value (kcal/100mL) (Mean ± SD)	Total	Weight	Mean IV, Random, 95%, CI	Forest Plot of Mean Caloric Density of Human Milk With 95% CI
9-12	Czosnykowska-Łukacka et al., 2018	65.76 ± 7.92	25	50.1%	65.76 [62.66; 68.86]	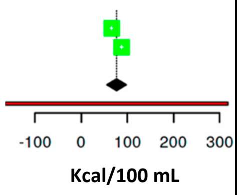
	Ongprasert et al., 2020	87.77 ± 13.61	47	49.9%	87.77 [83.88; 91.66]	
**Total (95% CI)**			**72**	100.0%	**76.73 [55.16; 98.30]**	
Heterogeneity: Tau^2 = 238.9950; Chi^2 = 75.11, df = 1 ($p < 0.0001$); I^2 = 98.7%						

Forest plot showing individual study estimates and pooled mean of breast milk caloric density (kcal/100 mL) in full-term infants aged 9–12 montha. Each green square represents the mean and 95% CI of a subgroup, with square size reflecting its weight in the meta-analysis. The black diamond represents the pooled estimate (random-effects model). The squares are ordered as in the table. Some studies contributed multiple data points due to different time points.
Abbreviations: SD: standard deviation; CI: confidence interval; IV: inverse variance.
Source: Based on data from Czosnykowska-Łukacka et al., 2018; Ongprasert et al., 2020.

Supplemental Table S13. Meta-analysis of caloric density of breast milk (kcal/100 mL) in full-term infants aged 12–18 month, based on systematic review data.

Age group (month)	Study	Individual value (kcal/100mL) (Mean ± SD)	Total	Weight	Mean IV, Random, 95%, CI	Forest Plot of Mean Caloric Density of Human Milk With 95% CI
**12-18**	Czosnykowska-Łukacka et al., 2018	78.34 ± 21.72	35	44.6%	78.34 [71.14; 85.54]	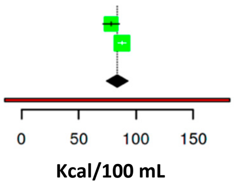
	Ongprasert et al., 2020	87.91 ± 13.23	50	55.4%	87.91 [84.24; 91.58]	
**Total (95% CI)**			**85**	100.0%	**83.65 [74.32; 92.97]**	
Heterogeneity: Tau² = 37.3027; Chi² = 5.39, df = 1 (p = 0.0202); I² = 81.5%						

Forest plot showing individual study estimates and pooled mean of breast milk caloric density (kcal/100 mL) in full-term infants aged 12–18 months. Each green square represents the mean and 95% CI of a subgroup, with square size reflecting its weight in the meta-analysis. The black diamond represents the pooled estimate (random-effects model). The squares are ordered as in the table. Some studies contributed multiple data points due to different time points.

Abbreviations: SD: standard deviation; CI: confidence interval; IV: inverse variance.

Source: Based on data from Czosnykowska-Łukacka et al., 2018; Ongprasert et al., 2020.

Supplemental Table S14. Meta-analysis of caloric density of breast milk (kcal/100 mL) in full-term infants aged 18–24 month, based on systematic review data.

Age group (month)	Study	Individual value (kcal/100mL) (Mean ± SD)	Total	Weight	Mean IV, Random, 95%, CI	Forest Plot of Mean Caloric Density of Human Milk With 95% CI
**18-24**	Czosnykowska-Łukacka et al., 2018	85.78 ± 20.07	41	47.5%	85.78 [79.64; 91.92]	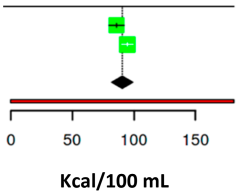
	Ongprasert et al., 2020	94.64 ± 16.13	44	52.5%	94.64 [89.87; 99.41]	
**Total (95% CI)**			**85**	100.0%	**90.43 [81.76; 99.10]**	
Heterogeneity: Tau² = 31.3810; Chi² = 4.99, df = 1 (p = 0.0255); I² = 80.0%						

Forest plot showing individual study estimates and pooled mean of breast milk caloric density (kcal/100 mL) in full-term infants aged 18–24 months. Each green square represents the mean and 95% CI of a subgroup, with square size reflecting its weight in the meta-analysis. The black diamond represents the pooled estimate (random-effects model). The squares are ordered as in the table. Some studies contributed multiple data points due to different time points.

Abbreviations: SD: standard deviation; CI: confidence interval; IV: inverse variance.

Source: Based on data from Czosnykowska-Łukacka et al., 2018; Ongprasert et al., 2020.
